# Infection avoidance behavior: Viral exposure reduces the motivation to forage in female *Drosophila melanogaster*

**DOI:** 10.1080/19336934.2016.1207029

**Published:** 2016-06-30

**Authors:** Pedro F. Vale, Michael D. Jardine

**Affiliations:** a Institute of Evolutionary Biology, School of Biological Sciences, University of Edinburgh, Edinburgh, UK; b Centre for Immunity, Infection and Evolution, University of Edinburgh, Edinburgh, UK

**Keywords:** avoidance behavior, Drosophila, DCV, foraging, Infection

## Abstract

Infection avoidance behaviors are the first line of defense against pathogenic encounters. Behavioral plasticity in response to internal or external cues of infection can therefore generate potentially significant heterogeneity in infection. We tested whether *Drosophila melanogaster* exhibits infection avoidance behavior, and whether this behavior is modified by prior exposure to Drosophila C Virus (DCV) and by the risk of DCV encounter. We examined 2 measures of infection avoidance: (1) the motivation to seek out food sources in the presence of an infection risk and (2) the preference to land on a clean food source over a potentially infectious source. While we found no evidence for preference of clean food sources over potentially infectious ones, previously exposed female flies showed lower motivation to pick a food source when presented with a risk of encountering DCV. We discuss the relevance of behavioral plasticity during foraging for host fitness and pathogen spread.

## Introduction

Hosts vary considerably in their ability to acquire and transmit infection,^1-3^ and much of this variation is caused by differences in the contact rate between susceptible individuals and sources of infection.^4,5^ For example, viruses of *Drosophila* fruit flies are not only widely distributed, they also show very broad host range.^6^ Given the high viral prevalence of pathogens in natural environments, mounting a timely and efficient immune response to all possible pathogenic challenges would be physiologically costly and ultimately ineffective. Hosts capable of reducing the probability of contacting parasites, infected conspecifics or infectious environments can therefore not only prevent the deleterious effects of infection, but also circumvent the undesirable energetic costs of immune responses, including immunopathology.^4,7^ Avoiding infection is therefore the first line of non-immunological defense against infection,^8^ and is known to occur across a broad range of host taxa.^7,9^

Like most traits, infection avoidance behaviors are likely to vary according to the context of infection, and pathogens are major drivers of this context.^4,7,9-11^ Pathogens may alter host responses in 2 ways. First, by altering the immunophysiology of the host during infection, pathogens can modify host behavior.^12,13^ Common behavioral changes in infected individuals include increased sleep and lethargy, or reduced feeding, mating, parental care or foraging (reviewed in^12,13^). In addition to internal, physiological cues of infection, external cues that indicate the magnitude of infection risk are also known to influence host behavioral responses.^4,7^ Understanding variation in infection avoidance behaviors therefore provides an important functional link between the neurological, behavioral and immunological processes that together govern the spread of disease.^12^

Insects are ideal systems to investigate the interplay between infection and behavior.^12,14^ The fruit fly *Drosophila* is especially amenable to these studies, as it is one of the best developed model systems for host-pathogen interactions^15^ and behavioral ecology and genetics.^16,17^ One of the most studied pathogenic interactions in *Drosophila* is the host response to systemic and enteric infection with Drosophila C Virus (DCV).^18,19^ DCV is a horizontally transmitted +ssRNA virus that naturally infects the fly gut,^19-21^ causing intestinal obstruction, severe metabolic dysfunction and eventually death.^22,23^ As a consequence of its pathology, female flies infected with DCV are also known to exhibit behavioral modifications, such as reduced locomotion and increased sleep.^24^ The Drosophila-DCV interaction therefore offers a powerful system to investigate the ecological consequences that may arise from the physiological and behavioral effects of enteric viral infections.

In the present study we used a combination of controlled experimental infections and foraging choice assays to test whether adult *D. melanogaster* are able to avoid potentially infectious environments, and if avoidance behavior is modified in response to virus exposure history and to different risks of acquiring DCV infection. We find evidence for avoidance behaviors in the form of reduced motivation to seek out and land on provided food sources according to the risk of infection. These effects were clearest in female flies previously exposed to DCV, indicating potentially important sexual dimorphism in infection avoidance.

## Results

**Figure 1.**
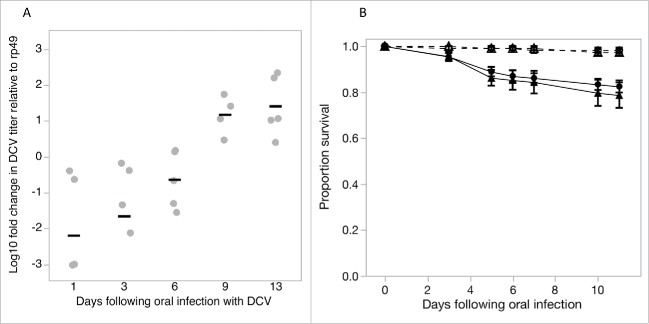
Exposing flies to DCV by placing them in DCV-contaminated vials for 3 d resulted in flies acquiring replicating virus as shown by the increase in DCV titres over time (1A). Gray points show the expression of DCV RNA titres relative to the expression of *rp49*, an internal fly control gene; black bars are mean titres (B). This orally acquired DCV infection had a moderate effect on fly survival in both male (full circle) and female (full triangle) flies compared to uninfected control male (open circle) and female (open triangle) flies (dashed lines). Data are means ± SEM.

Viral exposure prior to the behavioral assay was achieved by placing flies in a DCV contaminated environment for 3 days, allowing flies to acquire DCV infection orally. DCV acquired through the oral route using this protocol continued to replicate within the fly, increasing by 10–100 fold by day 13 following oral exposure (F_4,19_ = 8.78, p = 0.0003; Fig. 1A) and in both male and female flies resulted in up to 20% mortality within this period (Fig. 1B).

**Figure 2.**
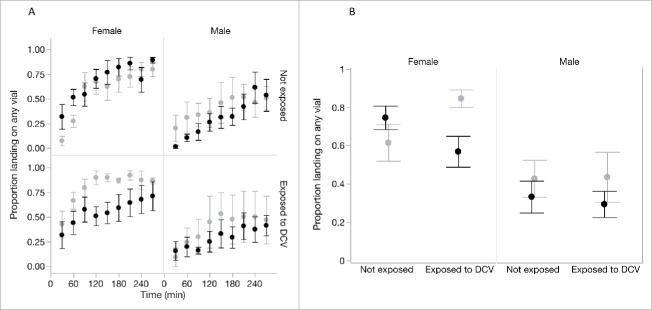
Single-sex groups of flies that had been previously exposed either to DCV or to a sterile Ringers solution were tested in a ‘no-risk’ environment (choice between 2 clean vials; light gray) or a ‘high-risk’ environment (choice between a clean vial and a DCV-contaminated vial; black). The motivation to seek out a food source, measured as the proportion of flies in the cage that landed on any of the provided food sources, increased over time (A). (B) shows the average motivation across the whole observation period for each combination of fly sex, prior DCV exposure and current exposure risk (‘no-risk’ environment (light gray) or a ‘high-risk’ environment (black). Data show means ± SEM.

To measure infection avoidance, we took 2 approaches. First, we hypothesized that the motivation to seek out food sources would be lower in environments where the risk of infection is higher.^7^ We therefore compared the proportion of flies that chose to seek out and land on any of the provided food sources in the “no risk” and “high-risk” cages. Only a fraction of flies chose either of the food sources provided, and this proportion increased over time for flies in all treatment groups (χ^2^_1_ = 11.00, p = 0.001; Fig. 2A). The rate at which motivation increased differed between sexes (‘Time × Sex' interaction, χ^2^_1_= 12.47, p=0.0004), and on average female flies showed greater motivation to forage than males (χ^2^_1_ = 5.01, p = 0.025), with 67% of female and 36% of male flies making a choice to land on any of the provided substrates during the observation period.

Across the entire observation period, the motivation to seek out and land on any of the provided food sources differed between sexes, and depended both on their previous exposure and on their current risk of infection (Fig 2B; ‘Sex’ × ‘risk of infection’ × ‘Previous exposure’ interaction, χ^2^_1_ = 21.82, p < 0.0001). The proportion of males choosing any food substrate did not vary with previous exposure to DCV in either high-risk (χ^2^_1_ = 2.21, p=0.137) or no-risk environments (χ^2^_1_ = 0.09, p = 0.764; Fig. 2).

In female flies however, previous exposure and current infection risk affected the motivation to land on the provided food sources. When there was no risk of infection (Fig. 2B, light gray bars) the motivation to seek out a food source was greater in females that were previously exposed to DCV than in otherwise healthy, non-exposed females (χ^2^_1_ = 104.11, p < 0.001). Among females that were previously exposed to infection, we found that the presence of a risk of acquiring infection resulted in lower foraging effort - with just over 50% of flies choosing a food source - compared to females in cages where there was no risk of acquiring infection, where over 80% of flies made the choice to land on a food source (Fig. 2B; χ^2^_1_ = 168.48, p < 0.001).

**Figure 3.**
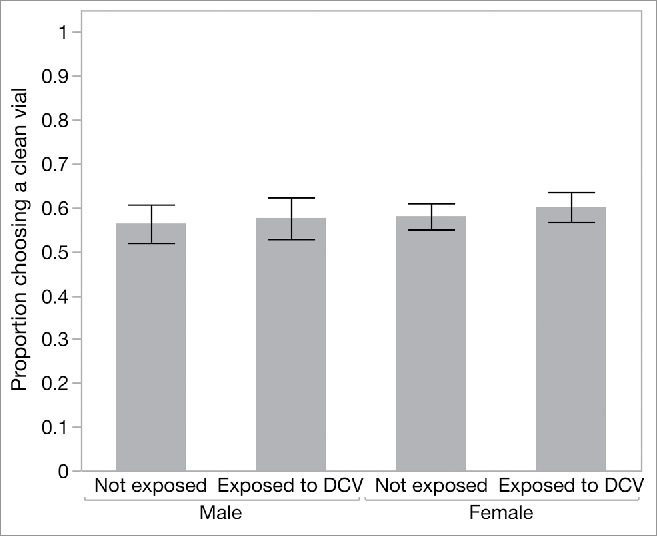
The proportion of flies in the high-risk cage that preferred to settle on the clean food source over the DCV-contaminated food source, according to sex and previous DCV exposure. Data are means ± SEM.

We also asked whether flies that chose to feed showed any evidence of avoiding potentially infectious food sources. For this analysis we focused on the “high risk” cages and recorded the proportion of flies choosing the clean food source over the infectious food source in each replicate cage. Once flies had made the choice to land on one of the provided food sources, the choice between a clean and a potentially infectious food source was not affected by previous exposure to DCV (‘previous exposure’, χ^2^_1_ = 0.513, p = 0.47) in either male or female flies (‘sex’, χ^2^_1_ = 0.595, p = 0.44) (Fig. 3).

## Discussion

The ability to detect and discriminate between clean and potentially infectious environments is vital to avoid the adverse consequences of infection. In this study we found that the motivation of female *Drosophila melanogaster* to seek out food sources is modified by its previous exposure to a viral pathogen and by the risk of encountering infection during foraging. Behavioral plasticity due to infection is widely reported among animals,^9,25^ and can be classified into (i) parasitic manipulation that enhances parasite transmission^9^ (ii) sickness behaviors that benefit the host by conserving energetic resources during infection,^13^ or (iii) side-effects of pathogenicity that do not benefit the host or the parasite.^25^

Female flies infected orally with DCV are known to experience increased lethargy and sleep,^24^ so these effects could also explain the reduced food seeking activity we detected in female flies that had been previously exposed to DCV. Another potential explanation for reduced motivation to find a food source in previously exposed flies is infection-induced anorexia,^26^ a commonly described sickness behavior.^13^ However, it is unlikely that lower motivation is simply a symptom of a “sick” fly, because in our experiment it varied according to the risk of infection, and even reached 80% in exposed flies when foraging in a ‘no risk’ environment (Fig. 2). This suggests that flies are actively avoiding contact with the potentially contagious food source by lowering their foraging effort.

The higher motivation of some female flies to seek out a food source when the risk of infection was absent (Fig. 2) suggests flies were able to identify external cues of infection risk. Identifying infection cues is a general prerequisite of avoidance behaviors and occurs across a wide range of different taxa. For example, lobsters are known to detect and avoid virus-infected conspecifics^27^; fruit flies and nematodes are capable of avoiding pathogenic bacteria^28,29^; gypsy moth larvae are able to detect and avoid virus-contaminated foliage^14^; sheep have been found to prefer to graze in parasite-poor patches^30^; and it is has been argued that the disgust response in humans has evolved because it decreases contact with potential infection.^31^ It is unclear how flies are able to identify food sources contaminated with a viral pathogen. In *Drosophila* and *C. elegans* avoidance of pathogenic bacteria is enabled by evolutionary conserved olfactory and chemosensory pathways,^28,29^ while avoidance of parasitic wasps appears to be mainly enabled by the visual sensory system.^32^ While avoiding virus infected conspecifics is probably driven by visual cues of infection,^27^ it remains unclear how virus-contaminated environments may trigger a lower motivation to feed in *Drosophila*.

The fact that only female flies demonstrated avoidance is an indication that any potentially adaptive effects of avoiding infection may be related to oviposition, which coincides with feeding. For flies previously exposed to DCV, avoiding infection would not confer substantial direct benefits given the physiological and behavioral costs of this infection,^22-24^ but would however reduce the exposure of future offspring to infection. While flies previously exposed to DCV do not appear to immune primed following an initial viral exposure,^33^ our results point to a sort of behavioral priming, where females previously exposed to infection avoid foraging in potentially infectious environments. Future work should therefore focus on testing how oviposition decisions are affected by female infection status and by external cues of infection. Oviposition decisions are likely to be critical for organismal fitness, especially if the ability of larvae to void infectious environments is reduced^32^

In summary, using a combination of experimental infections and behavioral assays, we find evidence that *Drosophila* can avoid infectious environments by showing reduced motivation to seek out a food source, which was most pronounced when flies were faced with an increased risk of encountering an infectious food source. However, these effects were only present in female flies, indicating potentially important sexual dimorphism in infection avoidance. Understanding how avoidance behaviors may vary is therefore important for our understanding of how disease will spread in natural populations,^4^ and more broadly how pathogens might evolve in response to variation in host infection avoidance strategies.^34,35^

## Materials and methods

### Fly and virus stocks

All flies used were from a long-term laboratory stock of Wolbachia-free *Drosophila melanogaster* Oregon R (OreR) line, maintained on Lewis medium in standard conditions: 25°C, with a 12:12 h light:dark cycle. Fly stocks were routinely kept on a 14-day cycle with non-overlapping generations under low larval densities. The DCV culture used in this experiment was grown in Schneider Drosophila Line 2 (DL2) as described in.^24^ Ten-fold serial dilutions of this culture (diluted in Ringers buffer solution) were aliquoted and frozen at −80°C for long-term storage before use.

### Virus exposure

**Figure 4.**
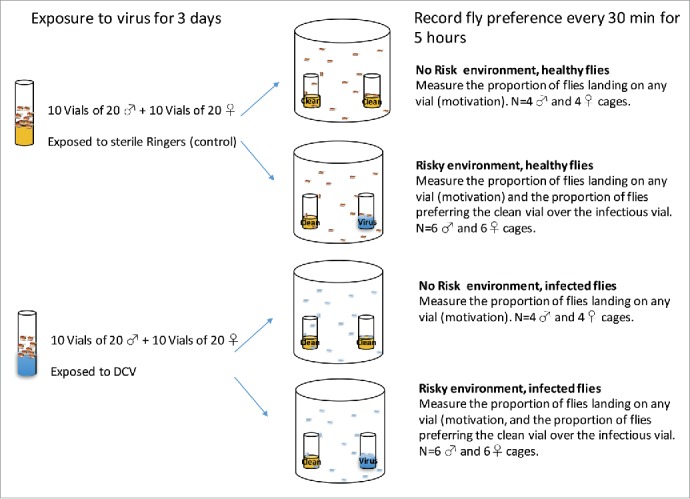
Schematic of the experimental setup.

Flies used in the foraging choice assays were obtained by preparing 10 vials of Lewis medium and yeast containing 10 mated females. Flies were allowed to lay eggs for 48 hours resulting in progeny reared in similar larval densities. To test the effect of previous exposure to virus on avoidance behavior during foraging, we exposed the progeny to DCV via the oral route of infection 2 to 3 d after eclosion. Oral DCV infection causes a small but significant reduction in fly survival^19^ and also experience changes in fecundity and fecal shedding (Vale, unpublished data), activity and sleep.^24^ Single-sex groups of 20 flies were placed in vials containing agar previously sprayed with DCV (“exposed” to 50 µl of 10^8^ viral copies/ml) or the equivalent volume of Ringers buffer solution as a control (“not exposed”). This procedure produced 10 replicate vials of either healthy or virus-exposed male or female flies (Fig. 4). The viral dose used here was lower than previously reported methods,^19^ so we first tested this dose was sufficient to result in viable DCV infections by measuring changes in virus titres and fly survival in separate experiments (Fig. 1). Fly survival was monitored on 9 replicate groups of 12 OreR flies per vial for 11 d following oral exposure. To measure changes in DCV titer, 2five, 2–3 day-old female flies were individually housed in vials previously sprayed with DCV as described above for 3 d. Five flies were collected 1, 3, 6, 9 or 13 d after exposure and total RNA was extracted from flies homogenized in Tri Reagent (Ambion), reverse-transcribed with M-MLV reverse transcriptase (Promega) and random hexamer primers, and then diluted 1:10 with nuclease free water. qRT-PCR was performed on an Applied Biosystems StepOnePlus system using Fast SYBR Green Master Mix (Applied Biosystems). We measured the relative fold change in DCV RNA relative to *rp49*, an internal *Drosophila* control gene, calculated as 2^−ΔΔCt^ as described in.^36^

### Foraging choice assays

Following 3 d of virus exposure, we set up independent foraging choice assays in cages - cylindrical transparent plastic containers (12 cm in diameter × 15 cm in height) containing 2 equally spaced plastic vials of standard Lewis fly medium supplemented with dry yeast (Fig. 4). For each combination of “DCV exposed” and “not exposed” male or female flies, we set up 2 sets of cages to simulate different risks of infection: a “no risk” environment, with 2 clean vials (sprayed with sterile Ringers solution), and a “high-risk” environment where one of the vials was sprayed with DCV, as described above. Six replicates of 20-fly groups were allocated to the “high-risk” chambers and 4 replicates to the “no risk” chambers, resulting in a total of 40 independent foraging choice cages (Fig. 4). Flies were transferred without anesthesia with the aid of an aspirator directly from vials into a neutrally placed hole in the lid of each chamber. The number of flies that settled on each vial was recorded every 30 minutes for 5 hours. Care was taken to randomize the position of the cages so that the orientation of the light did not influence the choice of the flies in any systematic way.

### Statistical analysis

In both analyses of ‘motivation to feed’ and ‘infection avoidance’, data on the proportion of flies choosing each food source within each replicate cage were analyzed with a generalized linear model assuming binomial error and logit link function, and included fly ‘sex’, ‘previous exposure’ and ‘infection risk’ as fixed effects. ‘Replicate cage’ was included as a random effect, nested within treatments. We also analyzed the average motivation to feed and infection avoidance across all time points, in a model that included “time” as a random effect. Treatment specific contrasts were used to test the significance of pairwise comparisons. Analyses were carried out using JMP 12.^37^
